# Genomic Confirmation of *Borrelia garinii*, United States

**DOI:** 10.3201/eid2901.220930

**Published:** 2023-01

**Authors:** Natalie Rudenko, Maryna Golovchenko, Ales Horak, Libor Grubhoffer, Emmanuel F. Mongodin, Claire M. Fraser, Weigang Qiu, Benjamin J. Luft, Richard G. Morgan, Sherwood R. Casjens, Steven E. Schutzer

**Affiliations:** Institute of Parasitology, Biology Centre Czech Academy of Sciences, Ceske Budejovice, Czech Republic (N. Rudenko, M. Golovchenko, A. Horak, L. Grubhoffer);; University of Maryland School of Medicine, Baltimore, Maryland, USA (E.F. Mongodin, C.M. Fraser);; Hunter College of the City University of New York, New York, New York, USA (W. Qiu);; Stony Brook University, Stony Brook, New York, USA (B.J. Luft);; New England Biolabs, Ipswich, Massachusetts, USA (R.G. Morgan);; University of Utah, Salt Lake City, Utah, USA (S.R. Casjens);; Rutgers New Jersey Medical School, Newark, New Jersey, USA (S.E. Schutzer)

**Keywords:** *Borrelia garinii*, bacteria, genomic conformation, migrating hosts, rodents, Lyme disease, vector-borne infections, zoonoses, United States

## Abstract

Lyme disease is a multisystem disorder primarily caused by *Borrelia burgdorferi* sensu lato. However, *B. garinii*, which has been identified on islands off the coast of Newfoundland and Labrador, Canada, is a cause of Lyme disease in Eurasia. We report isolation and whole-genome nucleotide sequencing of a *B. garinii* isolate from a cotton mouse (*Peromyscus gossypinus*) in South Carolina, USA. We identified a second *B. garinii* isolate from the same repository. Phylogenetic analysis does not associate these isolates with the previously described isolates of *B. garinii* from Canada.

Lyme disease is a multisystem disorder caused by infection with bacteria of the *Borrelia burgdorferi* sensu lato species complex. Three members of this complex, *B. burgdorferi* sensu stricto, *B. garinii*, and *B. afzelii*, are responsible for most cases of Lyme disease worldwide (*1*,*2*). *B. burgdorferi* s.l. is the only one of these 3 species that is found widely in North America, although *B. garinii* has been identified on islands off the coast of Newfoundland and Labrador, Canada (*3*–*5*).

We describe the isolation and genome sequencing characterization of a South Carolina *B. garinii* isolate from a repository of strains from rodent hosts and tick vectors in the southeastern United States that had been identified as *B. burgdorferi* s.l. A second *B. garinii* isolate from the same *B. burgdorferi* s.l. strain repository was identified on the basis of multilocus sequence typing (MLST). Phylogenetic analysis showed that these 2 strains from the southeastern United States were most closely related to a group of *B. garinii* isolates from Europe but were not derived from strains from Canada, or vice versa (*3*).

## Methods

### Sources, Cultivation, and Analyses of *Borrelia* spp.

The 2 *Borrelia* isolates described were isolated from ear biopsy specimens from a cotton mouse (*Peromyscus gossypinus*) (SCCH-7) trapped in Charleston County, South Carolina, in 1995 and from an eastern woodrat (*Neotoma floridana*) (SCGH-19) trapped in Georgetown County, South Carolina, in 1996 (Appendix) (*6*). We performed *Borrelia* culture in Barbour-Stoenner-Kelly H medium, DNA purification, and PCR analyses as described (*7*). We detected *B. burgdorferi* s.l. in samples by amplification of the 5S-23S intergenic region (*8*) (Appendix) by using species-specific PCR and primers designed on the basis of the *ospA* gene, which confirmed the presence of multiple spirochete species (*9*). Cultures in which *B. garinii* was confirmed were plated on solid medium, and clonal single colonies were isolated according to a modified protocol (*10*) (Appendix).

### Whole-Genome Sequencing and Genome Assembly

We performed whole-genome sequencing by using the Pacific Biosciences Sequel II system (https://www.pacb.com). We performed genome assembly by using the Genome Assembly tool in PacBio SMRTLink version 10.2 and 150 Mb of the HiFi reads >5 kb (Appendix).

### Nucleotide Sequence Accession Numbers

Sequences have been deposited in GenBank. The genome assembly of SCCH-7 has been deposited in GenBank under BioProject PRJNA431102 and BioSample accession no. SAMN26226110 (Appendix). Nucleotide sequences of 8 housekeeping genes (*clpA*, *clpX*, *nifS*, *pepX*, *pyrG*, *recG*, *rplB*, and *uvrA*) of SCCH-7 and SCGT-19 have been deposited in GenBank under accession nos. KP795353‒60 (SCCH-7) and KT285873‒80 (SCGT-19). The MLST sequences have also been deposited into the PubMLST database (https://pubmlst.org) under allele numbers assigned to unique loci (SCCH-7, *clpX* allele no. 272 and *uvrA* allele no. 278; SCGT-19, *clpA* allele no. 311 and *clpX* gene allele no. 273). Unique sequence type (ST) numbers in the PubMLST database are 1049 for SCCH-7 and 1050 for SCGT-19.

### Sequence Analysis

We performed MLST analysis of 8 housekeeping genes (*clpA*, *clpX*, *nifS*, *pepX*, *pyrG*, *recG*, *rplB*, and *uvrA*) of both isolates (SCCH-7 and SCGT-19) and whole-genome sequencing of strain SCCH-7 on DNA isolated at passage 6 as described (*11*). The maximum-likelihood phylogeny of *B. garinii* strains from a concatenated dataset of the 8 housekeeping loci sequences (184 total isolates, 4,791 nt) was inferred in RaxML (https://raxml-ng.vital-it.ch) under the generalized time reversible plus Γ4 model (Appendix). We performed phylogeographic analysis of diffusion on discrete space as implemented in BEAST (*12*) under the constant-size coalescent tree prior, and symmetric substitution model with Bayesian stochastic search variable selection enforced (Appendix). To compare sequences for the entire chromosome, we aligned the SCCH-7 chromosomal sequence with the 2 published chromosomal sequences of strain 20047 by using NUCMER (*13*). We derived a phylogenetic tree by using IQTREE (*14*) with default parameters from an MLST alignment of 34 *B. garinii* isolates most closely related to the 2 isolates from the United States.

## Results

### *B. garinii* from Rodents in South Carolina

The 2 *B. garinii* isolates we report were cultured from ear biopsy samples of a cotton mouse (*Peromyscus gossypinus*) (isolate SCCH-7) and an eastern woodrat (*Neotoma floridana*) (isolate SCGT-19); both were trapped in South Carolina (*6*). Those cultures were part of a southeastern United States collection of ≈300 *Borrelia* isolates that were obtained during 1991‒1999 in Missouri, Georgia, Florida, Texas, and South Carolina and housed in the James H. Oliver, Jr., Institute of Arthropodology and Parasitology, Georgia Southern University (Statesboro, Georgia, USA). Multiple *Borrelia* species in numerous cultures of this collection, often present as co-infections, were reported in earlier investigations, including *B. andersonii* (*15*–*18*), *B. burgdorferi* s.s. (*6*,*15*,*19*), *B. bissettiae* (*15*–*18*), *B. carolinensis* (*7*,*20*), *B. americana* (*21*), and a previously undescribed isolate from Texas, TXW-1 (*16*–*18*).

We confirmed *B. burgdorferi* s.l. in cultures by PCR amplification of total DNA with a 5S-23S rRNA set of primers (*8*). We identified the *B. burgdorferi* s.l. species present by cloning the total PCR products into the pCR4-TOPO TA vector and sequencing individual recombinants. We observed sequences with high similarity to *B. garinii* from 5 cultures. We then plated those cultures on solid medium to obtain single colonies and chose pure clonal cultures of *B. garinii* SCCH-7 clone 138 and SCGT-19 clone 19 from 2 of the cultures for further study.

### Whole-Genome Sequence of *B. garinii* Isolate SCCH-7

We determined the whole-genome sequence of isolate SCCH-7 by single-molecule real-time PacBio methods (Appendix). Similar to other *B. burgdorferi* sensu lato genomes (*22*–*24*), the SCCH-7 genome contains a linear chromosome and several linear plasmids (lp) and circular (cp) plasmids. SCCH-7 carries lp17, lp28–7, lp32–10, lp36, and lp54 and cp26, cp32–3, and cp32–6 (*25*). Plasmid SCCH-7 sequences are typical of known *B. garinii* genomes and are similar to those of *B. garinii* strain 20047, although strain 20047 carries an lp28–4 plasmid that is lacking in SCCH-7. The SCCH-7 genome is 1,161,212 bp (chromosome 906,106 bp, linear plasmids 168,083 bp, and circular plasmids 87,023 bp). Because the linear chromosome and plasmid sequences include all telomeres, this genome joins *B. burgdorferi* B31 and *B. mayonii* MN14–1539 genomes in being truly complete (*26*–*28*). The SCCH-7 chromosome differs from that of *B. garinii* strain 20047 by only 2 single-nucleotide variations (SNVs) and 2 short insertion/deletions from the sequence in accession no. CP028861 and by 8 SNVs and 4 short indels from the sequence in CP018744 (those 2 20047 chromosomal sequences were produced by 2 independent research groups, those of S. Bontemps-Gallo and G. Margos, Bioproject PRJNA224116). The 20047 plasmids were described briefly by Casjens et al. (*24*). This whole-genome sequence unambiguously demonstrates that SCCH-7 is a *B. garinii* isolate.

### Phylogenetic Analysis

*B. garinii* isolates from North America have been previously reported on coastal islands in the Atlantic Ocean in eastern Canada (*3*–*5*,*29*). To investigate whether the South Carolina isolates might have originated from these islands in Canada or vice versa, and to clarify the relationship of SCCH-7 and SCGT-19 with other *B. garinii* isolates, we amplified by using PCR and determined SCGT-19 sequences for the 8 genes (Appendix) previously used in MLST analyses of *B. burgdorferi* sensu lato isolates. We then extracted those sequences from the SCCH-7 and 20047 whole-genome sequences. 

We compiled a phylogenetic tree (Appendix Figure 1) of the MLST data from isolates in this branch of the *B. burgdorferi* s.l. species and a maximum-likelihood (RAxML) tree (Figure) of the MLST sequences that includes isolates SCCH-7 and SCGT-19 and the 178 other isolates available from the closely related species *B. garinii* and *B. bavariensis*, as well as 5 isolates of *B. turdi* as an outgroup (Appendix Table 1). Apart from 1 unusual isolate from European Russia (pubMLST ID:2488 Om16-103-Iapr) that is a sister branch to all the other isolates, the remaining 178 isolates form 2 clades that agree with previously defined *B. bavariensis* (39 isolates) and *B. garinii* (139 isolates) species (Figure) (*30*). The *B. bavariensis* group contains 4 isolates from Europe and 1 isolate from Canada, interspersed among most isolates from Asia, suggesting, on the basis of maximum parsimony, that this group is an ancestrally clade from Asia that has had several independent introgressions into Europe (*31*).

**Figure:**
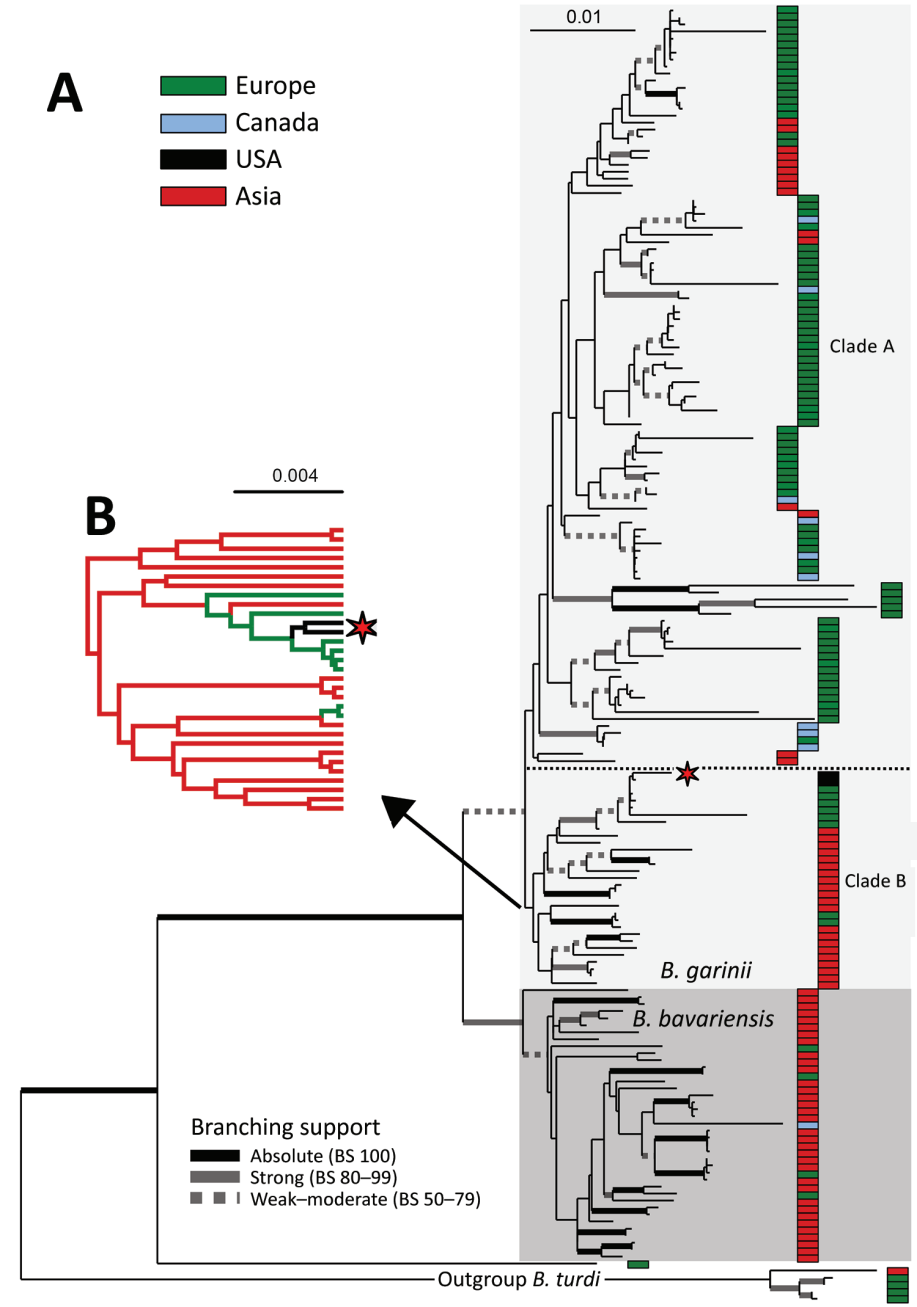
Maximum-likelihood phylogeny of *Borrelia garinii/B. bavariensis*. A) Maximum-likelihood phylogeny of *B. garinii/B. bavariensis* rooted with *B. turdi*. Topology is based on analysis of the partitioned dataset of 8 multilocus sequence typing genotyping loci s) under the generalized time reversible plus Γ4 model (for each partition) in RAxML 8 (https://cme.h-its.org/exelixis/web/software/raxml). The final alignment comprises 184 taxa and 4,791-nt positions. Thickened branches indicate branching support as estimated by nonparametric bootstrap analysis based on 1,000 replicates in RAxML 8. For better readability, support is categorized according to the scheme shown at the bottom of the tree. Isolates were clustered into 7 categories according to their geographic origin, which is color-coded according the scheme in the upper right part of the tree on the topology. The position of 2 US isolates is indicated by an asterisk. B) Subset of results phylogeographic analysis of diffusion on the discrete space showing the estimated geographic origin of the inner branches for the ancestral clade of *B. garinii* from Asia. Full topology is shown in Appendix Figure 2, panel B, and full details on the methods used are provided in the Appendix. Scale bars indicate nucleotide substitutions per site. BS, branching support.

The *B. garinii* clade is split into 2 major clades. The larger one (clade A, 108 isolates) comprises 76 isolates from Europe, 13 from continental Asia, 2 from Japan, 9 from Canada (Newfoundland and Labrador), and 8 from Iceland that are mostly distributed within this branch without apparent clustering by geographic origins. The smaller *B. garinii* clade (clade B, 31 isolates) contains 21 isolates from continental Asia and Japan, 8 from Europe, and the 2 described here from the United States. The 2 United States isolates form a nested subclade with 5 strains of European origin in clade B (Figure, panel B). Also, the *B. garinii* from Canada are members of clade A (Figure) and are not closely related to the United States isolates.

To shed more light on the possible origin of the 2 United States isolates and the evolutionary history of *B. garinii* in general, we performed phylogeographic analysis of diffusion in discrete space as implemented in BEAST (Appendix). This Bayesian method infers the ancestral state at each node of a given discrete trait (in this case, the geographic origin of the strain). The resulting tree topology (Appendix Figure 2, panel A), as inferred under the Coalescence (https://www2.unil.ch/popgen/softwares/quantinemo/coalescence.html) constant size model, separated the isolates into several groups. The *B. bavariensis* clade and clade B, whose composition corresponds to the MLST topology (Figure), are both predicted to originate in Asia. The 2 United States isolates (Figure) are in clade B and nested among a few sequences from Europe (Appendix Figure 2). The isolates from Europe are split into 3 clades (A1, A2, and A3), the first of which (A1) is found at the base of the whole *B. bavariensis/B. garinii* portion of the tree. Clade A1 is composed of 5 divergent *B. garinii* isolates from Slovakia separated from all other isolates from Europe and the *B. bavariensis* isolates. The ancestral clade from Asia (clade B) (Figure; Appendix Figure 2, panel A) is nested within clade A1 and clade A2, which were both predicted to have origins in Europe. The terminal position of strains from Europe in the ancestral clade from Asia (clade B) suggests a secondary, more recent introduction into Europe from Asia. Thus, ancestral reconstruction with BEAST suggested frequent historical and recent migration events of *B. bavarensis* and *B. garinii* species within Eurasia.

The affiliation of United States isolates with Europe and the broader *B. garinii* ancestral clade from Asia (clade B) is consistently present in both trees (Figure; Appendix Figure 2, panel A) and is supported by phylogeographic reconstruction using the *Bayesian stochastic search variable selection* algorithm (*32**–**34*). However, because of the large single number of isolates and relatively low number of phylogenetic-informative positions (i.e., high sequence similarity), the bootstrap support of inner branches was not high. Therefore, we tested the independent evolutionary history of isolates from the United States and Canada by using the approximately unbiased topology test (*35*). First, we force-constrained the monophyly of the 2 United States isolates with each of the 9 isolates from Canada; we then used RAxML to reoptimize the general topology. We then compared the per-site log-likelihood scores of those alternative topologies with the original MLST topology (Figure) by using the approximately unbiased in CONSEL (*36*). The resulting p values, ranging from 1.48 × 10^−36^ to 1.9 × 10^−2^ (Appendix Table 2), support the rejection of a common origin of *B. garinii* from Canada and the United States. We conclude that, in contrast to the isolates from Canada, which might have been introduced there from Europe or Iceland by seabirds and ticks associated with them (*37*), *B. garinii* from the southeastern United States are a part of ancestral lineage from East Asia that might have arrived in the United States from Europe.

### Chromosomal Relationships with Closely Related *B. garinii* Genomes

A maximum-likelihood tree of 32 closely related *B. garinii* genomes based on 8 housekeeping loci (Appendix Figure 3) shows that the 2 isolates from the United States cluster with a few isolates from Europe, which, by tree topology, were probably associated with an ancestor from Asia (Figure). We compiled all sequence differences at the 8 housekeeping loci among the strains that are most closely related to the 2 isolates from the United States (Appendix Table 3). The genome-derived MLST SCCH-7 sequence and 2 independent 20047 MLST sequences are nearly identical. The reported 20047 MSLT sequence has 2 differences in the *recG* gene compared with the 3 whole-genome sequences, probably caused by sequencing errors. Those sequence identities strongly support a non-Canada origin, specifically a recent Europe origin, of United States isolate SCCH-7.

In contrast to SCCH-7, strain SCGT-19 shows a distinct MLST haplotype defined by 16 SNVs and 1 short indel (Appendix Table 3). The SCGT-19 versions of some of these SNVs and the indel are found in other *B. garinii* strains from Japan and Europe, suggesting that they are unlikely to be sequencing errors. Furthermore, consecutive runs of SNVs at the *clpA* and *clpX* loci strongly indicate that their origins are caused by recombination and not de novo mutation. The differences between SCCH-7 and SCGT-19 suggests that a migration or importation of *B. garinii* from Eurasia to the United States might have consisted of multiple strains of a source population.

## Discussion

Our results provide strong evidence that *B. garinii* has been present in rodents in South Carolina, although its current status there is not known. Specifically, 5 samples we tested were positive for *B. garinii*, and from 2 independent *B. garinii* cultures, we propagated and analyzed, SCCH-7 clone 138 and SCCH-19 clone 19.

MLST analyses of both isolates and whole-genome sequencing of SCCH-7 showed that these isolates are not closely related to *B. garinii* strains from Canada; however, they are closely related to a subset of Eurasian isolates. How and when *B. garinii* arrived in South Carolina remains unknown. There were no reported Lyme disease outbreaks in the southeastern United States in humans at the time the strains were deposited in the repository or during the subsequent 2 decades. This finding minimizes the urgency for an immediate new search for *B. garinii* in this region. Nonetheless, clinical vigilance for *B. garinii* in humans in this region seems warranted.

Appendix Additional information on genomic confirmation of *Borrelia garinii*, United States.
