# Supplementary material for: Genomic Confirmation of Borrelia garinii, United States
Source: Emerg Infect Dis. 2023 Jan;29(1):64–9. doi: 10.3201/eid2901.220930 (PMC9796223; doi:10.3201/eid2901.220930)
Supplement: Appendix — Additional information on genomic confirmation of Borrelia garinii, United States. [file 22-0930-Techapp-s1.pdf]

Article DOI: https://doi.org/10.3201/eid2901.220930

Genomic Confirmation of *Borrelia garinii*, United States

Appendix

Rodents, Locations, and *Borrelia* Cultures

Borrelia cultures analyzed in this report were a part of collection of southeastern United States isolates. Two samples under investigation, SCCH-7 and SCGT-19, were isolated from the ear clip tissues of cotton mice (*Peromyscus gossypinus*) and eastern woodrat (*Neotoma floridana*), respectively. The ear clips represented a small triangular pieces of tissue from the very peripheral tip of the external pinna of each animal. Before the use of ear clip for culture seeding, the ear clips were cleaned with 95% ethanol, and tissues were sliced into smaller pieces, than washed once in 95% ethanol, followed by a rinse in a 1:1 mixture of 10% Clorox and 95% ethanol (*1*,*2*). *P. gossypinus* was trapped in Mt. Pleasant, Charleston County, South Carolina (USA) in 1995 and *N. floridana* was trapped in Georgetown County, South Carolina (USA) 1 year later. *Borrelia* isolates from the ear clip tissues were cultured in Barbour-Stoenner-Kelly (BSK) H medium that contained 0.15% agarose (Seakem; FMC Bioproducts, Rockland, ME), antimicrobial drugs (rifampin and phosphomycin), and fungicide (amphotericin B). The cultures were incubated in 5% CO_2 at 33°C–34°C. When the cultures reached a cell density of 2×10^6 spirochetes/mL, they were stored at −80°C (*3*).

Co-infection

The total DNA of all samples was purified with a DNeasy Blood and Tissue kit (QIAGEN) according to the manufacturer's recommendations. A *MasterTaq* Kit (Eppendorf, Germany) containing a special 5× TaqMaster PCR enhancer was used for the amplification of spirochete sequences. All PCRs were set up in a dedicated area, and precautions were taken to limit contamination (supplies, equipment and personal safety items, and pre- and post-amplification activities). In all steps of the analysis of reported samples, the DNA of *B. burgdorferi* sensu stricto strain B31 was used as a positive control. Initial PCR analysis of isolates from sample depository included amplification of the 5S–23S intergenic region (*4*), followed by sequencing. The cultures in which the presence of more than one spirochete species

was confirmed (overlapping chromatograms) were further analyzed by PCR amplification with species-specific *ospA*-targeting primers (*5*) that enabled detection of spirochetes from *B. burgdorferi* sensu lato complex (SL primers) and enabled differentiation of *B. burgdorferi* sensu stricto (GI primers, PCR product 543 bp), *B. garinii* (GII primers, PCR product 344 bp) and *B. afzelii* (GIII primers, PCR product 189 bp). PCR products of the expected size (344 bp) were cut out of the gel, purified with a Gel Extraction kit from QIAGEN, and sequenced in both directions with the same primer set as used for amplification. Further control of cultures that produced the GII amplicon (*B. garinii*) included amplification of the partial *flagellin* gene from the total DNA purified from co-infected culture, cloning of total PCR products into the pCR4-TOPO cloning vector, transformation of *Escherichia coli*, plating of recombinants into LB agar plates, picking of individual clones, and growth in 100 microliters of LB/ampicillin medium. Ninety-six well plates with recombinants were submitted for sequencing to the University of Washington (Seattle). Sequencing was conducted in both directions. The obtained sequences were used for similarity searching in GenBank. Co-infected cultures in which the presence of *B. garinii* was confirmed through the 2 above steps proceeded to separation of spirochete species by cultivation on solid BSK medium.

Nucleotide Sequence Accession Numbers

Sequences determined in this study have been deposited into GenBank with the following accession numbers for SCCH-7/SCGT-19, respectively: *flagellin* EU220774/EU220773; 5S–23S IGR–KP795350/KP795363; 16S-23S ITR–KP795352/KT285872; *ospA* KP795349/KP795362; *ospC* KP795351/KT285871 and *p66* KP795348/KP795361. The accession numbers for the eight housekeeping genes for SCCH-7/SCGT-19 are *clpA*, KP795360/KT285880; *clpX*, KP795358/KT285878; *nifS*, KP795357/KT285877; *pepX*, KP795359/KT285879; *pyrG*, KP795356/KT285876; *recG*, KP795355/KT285875; *rplB*, KP795353/KT285873; and *uvrA*, KP795354/KT285874. The genome assembly of SCCH-7 has been deposited in GenBank under BioProject PRJNA431102 with the BioSample accession SAMN26226110.

Unique loci detected in both South Carolina isolates were submitted to PubMLST database (https://pubmlst.org/) as well and were assigned unique alleles numbers as follows: isolate SCCH-7 *clpX* gene-allele number 272; *uvrA* gene- allele number 278. Isolate SCGT-19 *clpA* gene- allele number 311, *clpX* gene- allele number 273. Unique ST numbers in pubMLST database for SCCH-7/SCGT-19 are 1049 and 1050, respectively.

Whole-Genome Sequencing and Genome Assembly

Genome sequencing was performed by using the Pacific Biosciences Sequel II system. Total genomic DNA was isolated from SCCH-7 cells by using DNeasy Blood and Tissue Kit (QIAGEN) according to the manufacturer's instructions. The gDNA sample contained relatively short fragments mostly less than 5 kb, so no shearing was performed. A random library was prepared by using the PacBio SMRTbell express template kit 2.0 according to the manufacturer's instructions. Sequencing was performed by using one Sequel II cell which generated 178 Gb total sequence and 1.37M High Fidelity (HiFi) reads totaling 2.4Gb with mean length of 1757bp and mean quality of QV60. To facilitate assembly, the subset of HiFi reads longer than 5,000 bp was generated, which yielded 210-Mb sequence in 32.6 k reads with mean quality of QV42.

Genome assembly was performed with the Genome Assembly tool in PacBio SMRTLink 10.2 by using 150 Mb of the HiFi reads greater than 5kb (100× down sample with 1.5-Mb expected genome size). The assembly was polished by the PacBio Arrow algorithm, and the telomere ends were examined manually by comparison to multiple individual HiFi reads.

Phylogenetic Analysis

The sequences of 8 housekeeping loci (*clpA, clpX, nifS, pepX, pyrG, recG, rplB,* and *uvrA*) of *Borrelia* isolates were obtained from the PubMLST database (https://pubmlst.org/), and sequences of several additional relevant isolates were added from our collection. The resulting dataset was aligned with the MUSCLE aligner (*6*) implemented in SeaView 4 (*7*). To ensure the frame integrity of codon information, we translated the sequences into amino acids first, then aligned and back-translated them into nucleotides. Phylogenetic analysis with the maximum likelihood method was performed in RAxML (*8*) under the General time-reversible substitutional matrix, with nucleotide frequencies estimated from the dataset and four gamma-corrected rate site classes (GTR + G4 + F). The best topology and the branching support values were calculated by using the rapid bootstrap inferences from 1,000 replicates followed by a thorough ML search (fa parameter) in RAxML.

Phylogeographic analysis of diffusion on discrete space as implemented in BEAST (*9,10*) was performed under the GTR + G4 matrix, constant-size coalescent tree prior, and symmetric substitution model with BSSVS enforced. To make the analysis more feasible, we simplified the location coding into following geographic categories: Europe, Asia, United States and Canada.

To obtain sufficient effective sample sizes for the estimated parameters, we ran Monte Carlo Markov Chains (MCMC) for 100 million generations subsampling every 10,000 trees. The MCMC convergence was then inspected, and burnin value was selected in Tracer (*11*). The final topology, including the reconstruction of distribution of *B. garinii* strains was summarized in TreeAnnotator (*9*), and the topology was visualized in FigTree. The approximately unbiased test (*12*) as implemented in CONSEL (*13*) was used to test alternative phylogenetic topologies.

Prevalence of *B. garinii* in Samples from the Southeastern United States

During 2005–2017, we have studied a group of spirochete cultures that was a part of southeastern depository of *Borrelia burgdorferi* sensu lato strains in former James H. Oliver Institute of Arthropodology and Parasitology (Statesboro, Georgia, USA). Spirochetes from analyzed group were isolated from environmental samples, collected in 9 different localities of the southern region of the United States. Samples from the studied group were collected in South Carolina, Georgia, and Florida during 1993–1997. Spirochetes were cultured from 3 rodent species, cotton mouse *Peromyscus gossypinus,* eastern woodrat *Neotoma floridana*, and cotton rat *Sigmodon hispidus* (78 cultures), 3 tick species, *Ixodes affinis, I. minor*, and *I. scapularis* (40 cultures), and 9 bird species, Carolina wren (*Thryothorus ludovicianus*), Downy woodpecker (*Picoides pubescens*), White eyed vireo (*Vireo griseus*), Swainson’s thrush (*Catharus ustulatus*), American redstart (*Setophaga ruticilla*), Northern waterthrush (*Parkesia noveboracensis*), Pine warbler (*Setophaga pinus*), Northern cardinal (*Cardinalis cardinalis*), and Eastern towhee (*Pipilo erythrophthalmus*) (13 cultures) (Appendix Table 4).

Recultivation of spirochetes, DNA purification and PCR amplification of selected genomic loci were conducted as described above (*3*,*14*). The results of analyses revealed the presence of multiple spirochete species in the samples of the studied group: 16 strains represented new *Borrelia* species that were described as *Borrelia carolinensis* (*3*,*15*); another 7 isolates, that represented unknown spirochete species, were described as *Borrelia americana* (*16*,*17*); 53 isolates were identified as *Borrelia burgdorferi* sensu stricto (*14*,*18*); 36 cultures represented rather diverse group of *Borrelia bissettiae*. The remaining 19 isolates represented cultures with more than one spirochete species present (co-infected). All 13 cultures isolated from birds contained >1 spirochete species.

Of 131 cultures analyzed, *B. garinii* was detected by PCR amplification targeting the *ospA* gene with species-specific primers (*5*) in 5 co-infected cultures. The detected prevalence of *B. garinii* (3.8%) is based on results of the studied group only (not the whole collection of depository strains). Isolation of monoclonal populations of *B. garinii* was successful only for SCCH-7 and SCGT-19 isolates (Appendix Table 5).

References

1. Oliver JH Jr, Chandler FW Jr, Luttrell MP, James AM, Stallknecht DE, McGuire BS, et al. Isolation and transmission of the Lyme disease spirochete from the southeastern United States. Proc Natl Acad Sci U S A. 1993;90:7371–5. PubMed https://doi.org/10.1073/pnas.90.15.7371

2. Oliver JH Jr, Clark KL, Chandler FW Jr, Tao L, James AM, Banks CW, et al. Isolation, cultivation, and characterization of *Borrelia burgdorferi* from rodents and ticks in the Charleston area of South Carolina. J Clin Microbiol. 2000;38:120–4. PubMed https://doi.org/10.1128/JCM.38.1.120-124.2000

3. Rudenko N, Golovchenko M, Grubhoffer L, Oliver JH Jr. *Borrelia carolinensis* sp. nov., a new (14th) member of the *Borrelia burgdorferi* sensu lato complex from the southeastern region of the United States. J Clin Microbiol. 2009a;47:134–41. PubMed https://doi.org/10.1128/JCM.01183-08

4. Postic D, Assous MV, Grimont PA, Baranton G. Diversity of *Borrelia burgdorferi* sensu lato evidenced by restriction fragment length polymorphism of rrf (5S)-rrl (23S) intergenic spacer amplicons. Int J Syst Bacteriol. 1994;44:743–52. PubMed https://doi.org/10.1099/00207713-44-4-743

5. Demaerschalck I, Ben Messaoud A, De Kesel M, Hoyois B, Lobet Y, Hoet P, et al. Simultaneous presence of different *Borrelia burgdorferi* genospecies in biological fluids of Lyme disease patients. J Clin Microbiol. 1995;33:602–8. PubMed https://doi.org/10.1128/jcm.33.3.602-608.1995

6. Edgar RC. MUSCLE: multiple sequence alignment with high accuracy and high throughput. Nucleic Acids Res. 2004;32:1792–7. PubMed https://doi.org/10.1093/nar/gkh340

7. Gouy M, Guindon S, Gascuel O. SeaView version 4: A multiplatform graphical user interface for sequence alignment and phylogenetic tree building. Mol Biol Evol. 2010;27:221–4. PubMed https://doi.org/10.1093/molbev/msp259

8. Stamatakis A. RAxML version 8: a tool for phylogenetic analysis and post-analysis of large phylogenies. Mol Biol Evol. 2010;27:221–4.

9. Drummond AJ, Suchard MA, Xie D, Rambaut A. Bayesian phylogenetics with BEAUti and the BEAST 1.7. Mol Biol Evol. 2012;29:1969–73. PubMed https://doi.org/10.1093/molbev/mss075

10. Lemey P, Rambaut A, Drummond AJ, Suchard MA. Bayesian phylogeography finds its roots. PLOS Comput Biol. 2009;5:e1000520. PubMed https://doi.org/10.1371/journal.pcbi.1000520

11. Rambaut A, Drummond AJ. 2013. Tracer version 1. 5. http://beast.bio.ed.ac.uk.

12. Shimodaira H. An approximately unbiased test of phylogenetic tree selection. Syst Biol. 2002;51:492–508. PubMed https://doi.org/10.1080/10635150290069913

13. Shimodaira H, Hasegawa M. CONSEL: for assessing the confidence of phylogenetic tree selection. Bioinformatics. 2001;17:1246–7. PubMed https://doi.org/10.1093/bioinformatics/17.12.1246

14. Rudenko N, Golovchenko M, Hönig V, Mallátová N, Krbková L, Mikulásek P, et al. Detection of *Borrelia burgdorferi* sensu stricto *ospC* alleles associated with human Lyme borreliosis worldwide in non-human-biting tick *Ixodes affinis* and rodent hosts in southeastern United States. Appl Environ Microbiol. 2013a;79:1444–53. PubMed https://doi.org/10.1128/AEM.02749-12

15. Rudenko N, Golovchenko M, Grubhoffer L, Oliver JH Jr. *Borrelia carolinensis* sp. nov., a novel species of the *Borrelia burgdorferi* sensu lato complex isolated from rodents and a tick from the south-eastern USA. Int J Syst Evol Microbiol. 2011;61:381–3. PubMed https://doi.org/10.1099/ijs.0.021436-0

16. Rudenko N, Golovchenko M, Lin T, Gao L, Grubhoffer L, Oliver JH Jr. Delineation of a new species of the *Borrelia burgdorferi* sensu lato complex, *Borrelia americana* sp. nov. J Clin Microbiol. 2009b;47:3875–80. PubMed https://doi.org/10.1128/JCM.01050-09

17. Rudenko N, Golovchenko M, Belfiore NM, Grubhoffer L, Oliver JH Jr. Divergence of *Borrelia burgdorferi* sensu lato spirochetes could be driven by the host: diversity of *Borrelia* strains isolated from ticks feeding on a single bird. Parasit Vectors. 2014;7:4. PubMed https://doi.org/10.1186/1756-3305-7-4

18. Rudenko N, Golovchenko M, Grubhoffer L, Oliver JH Jr. The rare *ospC* allele L of *Borrelia burgdorferi* sensu stricto, commonly found among samples collected in a coastal plain area of the southeastern United States, is associated with *ixodes affinis* ticks and local rodent hosts *Peromyscus gossypinus* and *Sigmodon hispidus.* Appl Environ Microbiol. 2013b;79:1403–6.2 PubMed https://doi.org/10.1128/AEM.03362-12

19. Margos G, Gatewood AG, Aanensen DM, Hanincová K, Terekhova D, Vollmer SA, et al. MLST of housekeeping genes captures geographic population structure and suggests a European origin of *Borrelia burgdorferi.* Proc Natl Acad Sci U S A. 2008;105:8730–5. PubMed https://doi.org/10.1073/pnas.0800323105

Appendix Table 1. *Borrelia* strains included into phylogenetic analysis based on 8 housekeeping genes*

pubMLST ID	Strain name	Sample location	Species
62	164/11g	Serbia	*Borrelia garinii*
153	20047	France	*Borrelia garinii*
166	IPT140	France	*Borrelia garinii*
170	IPT130	France	*Borrelia garinii*
171	IPT139	France	*Borrelia garinii*
172	IPT165	France	*Borrelia garinii*
193	MDH1	China	*Borrelia garinii*
206	VH4	China	*Borrelia garinii*
211	VL2	China	*Borrelia garinii*
271	70576B	UK	*Borrelia garinii*
279	6910BT	UK	*Borrelia garinii*
284	61030BT	UK	*Borrelia garinii*
290	70531B	UK	*Borrelia garinii*
349	62303L	Latvia	*Borrelia garinii*
351	61209L	Latvia	*Borrelia garinii*
372	75803L	Latvia	*Borrelia garinii*
476	80201G	Germany	*Borrelia garinii*
513	82805G	Germany	*Borrelia garinii*
1041	TS1	UK	*Borrelia garinii*
1042	DR46	UK	*Borrelia garinii*
1045	LV5M	UK	*Borrelia garinii*
1046	DV12	UK	*Borrelia garinii*
1047	b34	UK	*Borrelia garinii*
1048	DV46	UK	*Borrelia garinii*
1050	DV13	UK	*Borrelia garinii*
1054	LA9	UK	*Borrelia garinii*
1055	LA34	UK	*Borrelia garinii*
1062	MNG61	Mongolia	*Borrelia garinii*
1077	HkIP2	Japan	*Borrelia garinii*
1078	HP1	Japan	*Borrelia garinii*
1079	HP3	Japan	*Borrelia garinii*
1083	NP4	Japan	*Borrelia garinii*
1085	NP76	Japan	*Borrelia garinii*
1086	NP81	Japan	*Borrelia garinii*
1088	NT25	Japan	*Borrelia garinii*
1089	NT31	Japan	*Borrelia garinii*
1091	Ip-90	Russia	*Borrelia garinii*
1093	Np189	Russia	*Borrelia garinii*
1128	J-37 (human)	Japan	*Borrelia garinii*
1137	0510578I	Italy	*Borrelia garinii*
1138	0510605I	Italy	*Borrelia garinii*
1172	0220354I	Italy	*Borrelia garinii*
1323	rebun_20	Japan	*Borrelia garinii*
1328	konnai_1	Japan	*Borrelia garinii*
1334	konnai_15_clone5	Japan	*Borrelia garinii*
1345	EU9-22	The Netherlands	*Borrelia garinii*
1426	Ekb701-11	Russia	*Borrelia garinii*
1429	Ekb712-11	Russia	*Borrelia garinii*
1478	akt7	Norway	*Borrelia garinii*
1480	akt10	Norway	*Borrelia garinii*
1481	akt11	Norway	*Borrelia garinii*
1487	akt19	Norway	*Borrelia garinii*
1509	akt43	Norway	*Borrelia garinii*
1512	akt46	Norway	*Borrelia garinii*
1518	akt52	Norway	*Borrelia garinii*
1519	akt53	Norway	*Borrelia garinii*

pubMLST ID	Strain name	Sample location	Species
1707	PKuf (human)	Germany	*Borrelia garinii*
1743	Ekb151-2012	Russia	*Borrelia garinii*
1758	Ekb783-2013	Russia	*Borrelia garinii*
1770	Nsk1400-2013	Russia	*Borrelia garinii*
1790	Tmsk1125-2013	Russia	*Borrelia garinii*
1791	Tmsk1128-2013	Russia	*Borrelia garinii*
1792	Tmsk1130-2013	Russia	*Borrelia garinii*
1793	Tmsk1187-2013	Russia	*Borrelia garinii*
1794	Tmsk1188-2013	Russia	*Borrelia garinii*
1795	Tmsk1189-2013	Russia	*Borrelia garinii*
1798	Tmsk1193-2013	Russia	*Borrelia garinii*
1800	Tmsk1218-2013	Russia	*Borrelia garinii*
1808	Tmsk2148-2014	Russia	*Borrelia garinii*
1824	PCoo (human)	Germany	*Borrelia garinii*
1826	PMag (human)	Germany	*Borrelia garinii*
1827	PMek (human)	Germany	*Borrelia garinii*
1829	PUl (human)	Germany	*Borrelia garinii*
1860	Tom5202	Russia	*Borrelia garinii*
1863	Tom2903	Russia	*Borrelia garinii*
1864	Tom1805	Russia	*Borrelia garinii*
1866	Tom8705	Russia	*Borrelia garinii*
1901	NL11-021	Canada	*Borrelia garinii*
1904	NL12-114	Canada	*Borrelia garinii*
1910	NL12-334C	Canada	*Borrelia garinii*
1911	NL12-340F	Canada	*Borrelia garinii*
1913	NL13-029	Canada	*Borrelia garinii*
1918	NL13-245	Canada	*Borrelia garinii*
1919	NL13-440	Canada	*Borrelia garinii*
1921	NL13-534	Canada	*Borrelia garinii*
1923	NL14-1000	Canada	*Borrelia garinii*
1962	PBe (human)	Germany	*Borrelia garinii*
1981	PNel (human)	Germany	*Borrelia garinii*
1992	PStg (human)	Germany	*Borrelia garinii*
2034	HN13	South Korea	*Borrelia garinii*
2452	EuTu104	Czech Republic	*Borrelia garinii*
2454	EuTu185	Czech Republic	*Borrelia garinii*
2457	EuTu251	Slovenia	*Borrelia garinii*
2459	EuTu347	Sweden	*Borrelia garinii*
2460	EuTu352	Sweden	*Borrelia garinii*
2463	EuTu451	The Netherlands	*Borrelia garinii*
2467	EuTu490	Finland	*Borrelia garinii*
2471	EuTu488	Finland	*Borrelia garinii*
2473	EuTu456	Finland	*Borrelia garinii*
2475	EuTu519	Estonia	*Borrelia garinii*
2479	EuTu476	Finland	*Borrelia garinii*
2515	DNQ100	Slovakia	*Borrelia garinii*
2523	DNQ9	Slovakia	*Borrelia garinii*
2583	451_UA	Ukraine	*Borrelia garinii*
2669	132DIVN1	Slovakia	*Borrelia garinii*
2698	NE5245	Switzerland	*Borrelia garinii*
2756	17-58N4_wgs	Norway	*Borrelia garinii*
2875	85DIVN13	Slovakia	*Borrelia garinii*
2906	9-22-27	Latvia	*Borrelia garinii*
3068	8/1/2029	Latvia	*Borrelia garinii*
3070	1-31LT	Latvia	*Borrelia garinii*
3083	2-27LT	Latvia	*Borrelia garinii*
3107	3-17-10	Latvia	*Borrelia garinii*
3113	10-26-29	Latvia	*Borrelia garinii*
3140	NE4906	Switzerland	*Borrelia garinii*
3177	MaN25417/86	Slovakia	*Borrelia garinii*
3178	MaN25417/149	Slovakia	*Borrelia garinii*
3179	MaN111017/219	Slovakia	*Borrelia garinii*
3181	KF3517/45	Slovakia	*Borrelia garinii*
3182	KF3517/47	Slovakia	*Borrelia garinii*
3184	ZSF25417/69	Slovakia	*Borrelia garinii*
NA	NLD124_2011	The Netherlands	*Borrelia garinii*
NA	NLD128_2011	The Netherlands	*Borrelia garinii*
NA	NLD132_2010	The Netherlands	*Borrelia garinii*
NA	NLD135_2011	The Netherlands	*Borrelia garinii*

pubMLST ID	Strain name	Sample location	Species
NA	NLD145_2010	The Netherlands	*Borrelia garinii*
NA	NLD146_2010	The Netherlands	*Borrelia garinii*
NA	NLD149_2010	The Netherlands	*Borrelia garinii*
NA	NLD212_2009	The Netherlands	*Borrelia garinii*
1049	USA233_1995	USA	*Borrelia garinii*
1050	USA234_1996	USA	*Borrelia garinii*
NA	ISL367_2010	Iceland	*Borrelia garinii*
NA	ISL369_2010	Iceland	*Borrelia garinii*
NA	ISL370_2010	Iceland	*Borrelia garinii*
NA	ISL371_2010	Iceland	*Borrelia garinii*
NA	ISL372_2010	Iceland	*Borrelia garinii*
NA	ISL373_2010	Iceland	*Borrelia garinii*
NA	ISL375_2010	Iceland	*Borrelia garinii*
NA	ISL376_2010	Iceland	*Borrelia garinii*
198	NMK6	China	*Borrelia bavariensis*
202	JW3	China	*Borrelia bavariensis*
203	VH1	China	*Borrelia bavariensis*
204	VH2	China	*Borrelia bavariensis*
205	VH3	China	*Borrelia bavariensis*
209	VH19	China	*Borrelia bavariensis*
214	JLHCH	China	*Borrelia bavariensis*
216	HQ	China	*Borrelia bavariensis*
1060	MNG14	Mongolia	*Borrelia bavariensis*
1061	MNG24	Mongolia	*Borrelia bavariensis*
1076	HkIP1	Japan	*Borrelia bavariensis*
1082	N346	Japan	*Borrelia bavariensis*
1087	NT24	Japan	*Borrelia bavariensis*
1092	Mp7	Russia	*Borrelia bavariensis*
1095	HkCR3	Japan	*Borrelia bavariensis*
1104	FsAE1	Japan	*Borrelia bavariensis*
1112	ChYAE2	China	*Borrelia bavariensis*
1118	J-15	Japan	*Borrelia bavariensis*
1119	J-16	Japan	*Borrelia bavariensis*
1122	J-20T	Japan	*Borrelia bavariensis*
1134	HH1	Japan	*Borrelia bavariensis*
1313	enkichi_32	China	*Borrelia bavariensis*
1333	konnai_14	Japan	*Borrelia bavariensis*
1340	takamine_As_5	Japan	*Borrelia bavariensis*
1431	Prm7564-11	Russia	*Borrelia bavariensis*
1440	Ekb166-10	Russia	*Borrelia bavariensis*
1442	Alt763-11	Russia	*Borrelia bavariensis*
1446	Arh976-12	Russia	*Borrelia bavariensis*
1459	PScf	Germany	*Borrelia bavariensis*
1735	Arh913-2012	Russia	*Borrelia bavariensis*
1741	Ekb1421-2014	Russia	*Borrelia bavariensis*
1745	Ekb169-2012	Russia	*Borrelia bavariensis*
1802	Tmsk1253-2013	Russia	*Borrelia bavariensis*
1804	Tmsk1613-2014	Russia	*Borrelia bavariensis*
1839	Mng4702	Mongolia	*Borrelia bavariensis*
1843	Tom1003	Russia	*Borrelia bavariensis*
1853	Tom4606	Russia	*Borrelia bavariensis*
1859	Tom5007	Russia	*Borrelia bavariensis*
1902	NL11-061	Canada	*Borrelia bavariensis*
2488	Om16-103-Iapr	Russia	*Borrelia sp.*
1283	PoTiBtur10	Portugal	*Borrelia turdi*
1285	PoTiBtur12	Portugal	*Borrelia turdi*
1458	Ya501	Japan	*Borrelia turdi*
2075	T2084	Portugal	*Borrelia turdi*
2076	TPT2017	Portugal	*Borrelia turdi*

*NA, not available.

Appendix Table 2. Results of approximately-unbiased (au) topology test comparing the original most-likely topology as seen on Figure 1 (row 1), with the alternative topologies with enforced Canadian-US *B. garinii* monophyly (rows 2–11). The p-AU column denotes the statistical significance (p values) of the test for individual topology

Tree	logL	deltaL	p-AU
1	−22917.7	0	0.995
2	−23054.4	136.65	0.0002
3	−23090.9	173.17	0.000748
4	−23143.6	225.85	6.44E-05
5	−23201.7	283.95	1.48E-36
6	−23030.6	112.85	0.0192
7	−23181.5	263.79	3.62E-07
8	−23159.1	241.34	0.000139
9	−23099.5	181.79	0.00125
10	−23051.1	133.35	0.00791
11	−23055.2	137.41	0.00345

Appendix Table 3. Distribution of single nucleotide variants (SNVs) among the housekeeping genes

MLST locus/position*			*clpA* (579 nt)							*clpX* (624 nt)					*nifS*(564nt)				*pyrG* (603 nt)						*recG* (651 nt)					*rplB* (624 nt)					*uvrA* (570 nt)				
Strain	Origin	ID	57	201	207	342	355	413	444	192	342	382-384	548	558	13	336	425	447	78	405	468	477	524	585	249	303	351	474	546	15	69	420	468	585	267	330	370	477	498
20047	France	CP028861	A	A	C	T	A	C	C	C	A	---	A	T	A	A	A	G	T	C	C	T	G	A	T	T	T	G	T	A	A	G	A	A	T	T	G	T	C
20047	France	CP018744	A	A	C	T	A	C	C	C	A	---	A	T	A	A	A	G	T	C	C	T	G	A	T	T	T	G	T	A	A	G	A	A	T	T	G	T	C
20047	France	153	A	A	C	T	A	C	C	C	A	---	A	T	A	A	A	G	T	C	C	T	G	A	T	T	T	G	T	A	A	G	A	A	T	T	G	T	C
PCoo	Germany	1824	A	A	C	T	A	C	C	C	A	---	A	T	A	A	A	G	T	C	C	T	G	A	T	T	T	G	T	A	A	G	A	A	T	T	G	T	C
DNQ100	Slovakia	2515	A	A	C	T	A	C	C	C	A	---	A	T	A	A	A	G	T	C	C	T	G	A	T	T	T	G	T	A	A	G	A	A	T	T	G	T	C
1-31LT	Latvia	3070	A	A	C	T	A	C	C	C	A	---	A	T	A	A	A	G	T	C	C	T	G	A	T	T	T	G	T	A	A	G	A	A	T	T	G	T	C
SCCH-7**	USA	1049	A	A	C	T	A	C	C	C	A	AGA	A	T	A	A	A	G	T	C	C	T	G	A	T	T	T	G	T	A	A	G	A	A	T	T	G	T	C
SCGT-19	USA	1050	A	G	C	C	G	C	C	C	A	AGA	G	C	A	A	A	G	T	C	C	T	G	A	T	T	T	G	T	A	A	G	A	A	T	T	G	T	C
HP1	Japan	1078	A	A	C	T	A	C	C	C	A	---	A	T	A	A	A	G	C	T	T	C	G	G	T	T	T	G	T	A	A	G	A	A	T	C	A	C	T
NT31	Japan	1089	A	A	C	T	A	C	C	C	A	AGA	G	C	C	A	A	G	T	T	C	T	G	G	C	C	C	A	C	G	G	A	G	G	T	C	A	C	T
rubun20	Japan	1323	A	A	C	T	A	C	C	A	G	---	G	C	C	A	A	G	C	T	T	C	G	G	C	C	C	A	C	G	G	A	G	G	T	C	A	C	T
NL11-021	Canada	1901	G	G	T	C	G	C	T	A	G	AGA	G	C	C	G	G	A	C	T	T	C	A	G	C	C	C	A	C	A	G	A	G	G	T	C	A	T	T
NL12-334C	Canada	1910	A	G	C	C	G	T	T	A	G	AGA	G	C	C	G	G	A	C	T	T	C	A	G	C	C	C	A	C	G	G	A	G	G	C	C	A	C	T
NL12-340F	Canada	1911	A	G	C	C	G	T	T	A	G	AGA	G	C	C	G	G	A	C	T	T	C	A	G	C	C	C	A	C	G	G	A	G	G	C	C	A	C	T
NL13-029	Canada	1913	A	G	C	C	G	T	T	A	G	AGA	G	C	C	G	G	A	C	T	T	C	A	G	C	C	C	A	C	G	G	A	G	G	C	C	A	C	T
NL13-245	Canada	1918	G	G	T	C	G	T	T	C	A	AGA	G	C	C	A	A	G	C	T	C	T	G	G	C	C	C	A	C	A	G	A	G	A	C	C	A	C	T
NL13-440	Canada	1919	G	G	T	C	G	C	T	A	G	AGA	G	C	C	G	G	A	C	T	T	C	A	G	C	C	C	A	C	A	G	A	G	G	T	C	A	T	T
NL13-534	Canada	1921	G	G	T	C	G	C	T	C	A	AGA	G	C	C	G	G	A	C	T	T	C	A	G	C	C	C	A	C	A	G	A	G	G	T	C	A	T	T
NL14-1000	Canada	1923	G	G	T	C	G	T	T	C	A	---	A	C	C	G	G	A	C	T	T	C	A	G	C	C	C	A	C	A	A	A	A	A	C	C	A	C	T

*Position of SNP conflicts is shown on respected housekeeping genes, which size was adjusted according to the MLST scheme (*19*).

**Sequences of respective genes of strain SCCH-7 were extracted from genome sequences.

Appendix Table 4. Composition of the analyzed group of samples from strains depository, United States

Location	Σ	*P. gossypinus*	*N. floridana*	*S. hispidus*	*I. scapularis*	*I. affinis*	*I. minor*	Birds
Georgia	21	9	4	2	0	6	0	0
Florida	3	1	0	2	0	0	0	0
South Carolina	94	32	20	8	4	18	12	13
Total	118	42	24	12	4	24	12	13
			Rodents 78			Ticks 40		

Appendix Table 5. Sample description and current state of *B. garinii*-positive cultures, detected in strains depository.

Sample	Collection date/site	Host	Source	Culture	Original status	Present status
SCCH-7	February 1995/SC USA	Cotton mouse	Ear clip	Cultured	Co-infection	Monoclonal
SCGT-19	January 1996/SC USA	Eastern woodrat	Ear clip	Cultured	Co-infection	Monoclonal
SCI-8	November 1997/SC USA	Downy woodpecker	Skin	Cultured	Co-infection	Co-infection
BUL-12	November 1997/GA USA	Cotton rat	Ear clip	Cultured	Co-infection	Co-infection
GAC-6	January 1998/GA USA	Cotton mouse	Ear clip	Cultured	Co-infection	Co-infection

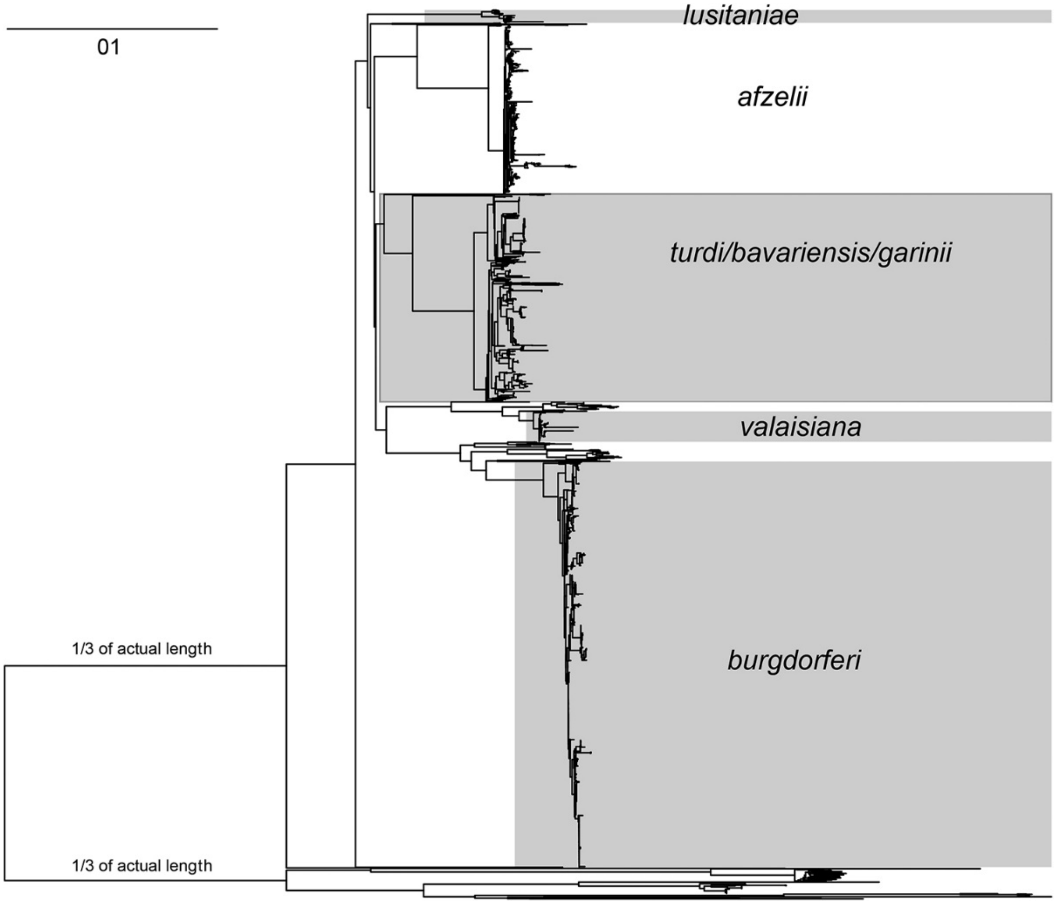

Appendix Figure 1. Unrooted maximum likelihood phylogeny of *Borrelia* based on analysis of 8 MLST genotyping loci (see above for details) under the GTR + G4 model implemented in RAxML 8. The final alignment comprises 2,977 taxa and 4,815- nt positions. Length of basal branches was reduced for formatting reasons.

A

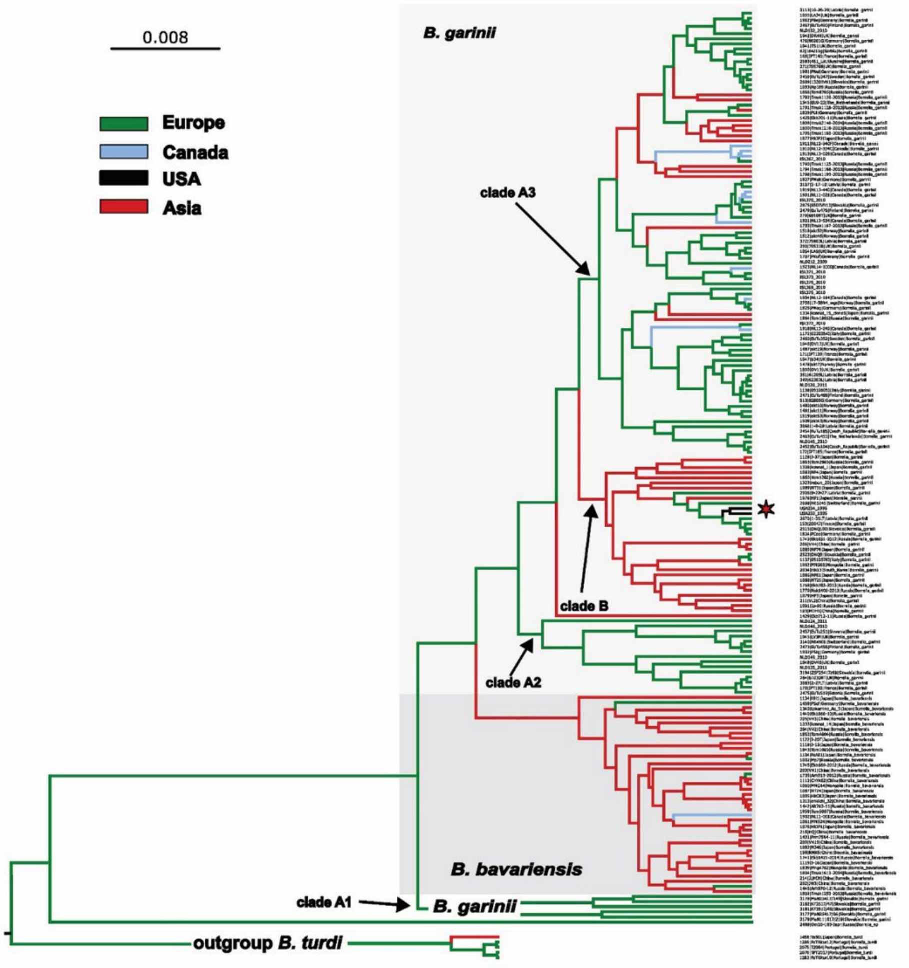
0.008
B. garinii
Europe
Canada
USA
Asia
clade A3
clade B
clade A2
B. bavariensis
clade A1
B. garinii
outgroup B. turdi

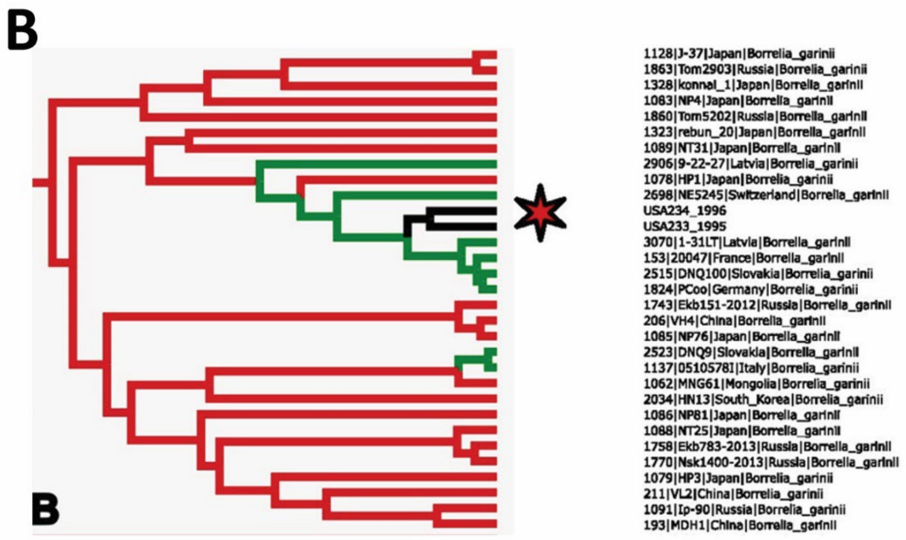

Appendix Figure 2. A) Phylogeographic analysis of *B. garinii/B. bavariensis* rooted with *B. turdi* by using the diffusion on the discrete space algorithm implemented in BEAST under the GTR + G4 matrix, constant-size coalescent tree prior, and symmetric substitution model with BSSVS enforced. The isolates were clustered into 4 categories according to their geographic origin, coded in form of branch colors according the scheme in the upper right part of the tree on the topology. The position of two USA isolates is indicated by an asterisk. B) Ancestrally Asian clade. Maximum-likelihood phylogeny of *B. garinii* based on analysis of the partitioned dataset of 8 MLST genotyping loci (see Methods for details) under the GTR + G4 model (for each partition) in RAxML 8. Subset of results of diffusion on the discrete space showing the estimated geographic origin of the inner branches for the clade, on which USA-originated *B. garinii* were allocated. *B. garinii* type strain 20047 is indicated by the arrow. Full topology is shown in panel A.

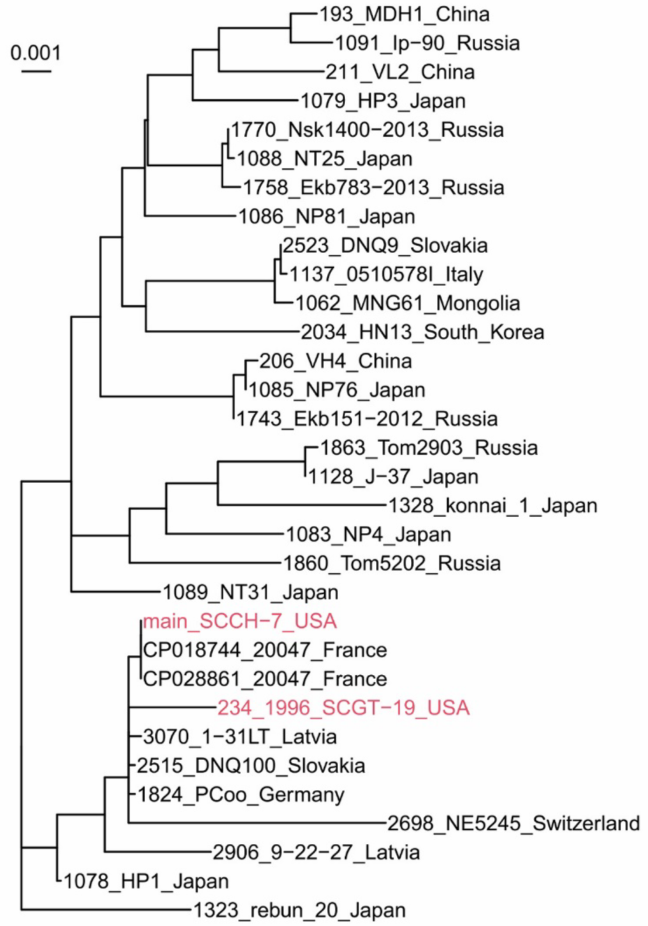

Appendix Figure 3. Maximum-likelihood tree of 32 closely related *B. garinii* isolates based on sequences at the 8 housekeeping loci. The maximum-likelihood tree was inferred with IQTREE. All branches shown are supported by a bootstrap value >80%. Two US isolates of *B. garinii* are highlighted in red. The SCCH-7 MLST sequence, grouped with the 2 previously sequenced genomes of strain 20047 (CP028861 and CP018744), was derived from the genome sequence.
